# Evaluation of inter-day and inter-individual variability of tear peptide/protein profiles by MALDI-TOF MS analyses

**Published:** 2012-06-14

**Authors:** Nerea González, Ibon Iloro, Juan A. Durán, Félix Elortza, Tatiana Suárez

**Affiliations:** 1 Bioftalmik, Parque Tecnológico de Vizcaya, Derio, Spain; 2 Proteomics Platform, CIC bioGUNE, CIBERehd, ProteoRed-ISCIII, Parque Tecnológico de Vizcaya, Derio, Spain; 3 Instituto Clínico Quirúrgico de Oftalmología (ICQO), Bilbao, Spain; 4 Department of Ophthalmology, School of Medicine, University of Basque Country, Spain

## Abstract

**Purpose:**

To characterize the tear film peptidome and low molecular weight protein profiles of healthy control individuals, and to evaluate changes due to day-to-day and individual variation and tear collection methods, by using solid phase extraction coupled to matrix-assisted laser desorption/ionization time-of-flight mass spectrometry (MALDI-TOF MS) profiling.

**Methods:**

The tear protein profiles of six healthy volunteers were analyzed over seven days and inter-day and inter-individual variability was evaluated. The bilaterality of tear film and the effect of tear collection methods on protein profiles were also analyzed in some of these patients. MALDI-TOF MS analyses were performed on tear samples purified by using a solid phase extraction (SPE) method based on C18 functionalized magnetic beads for peptide and low molecular weight protein enrichment, focusing spectra acquisition on the 1 to 20 kDa range. Spectra were analyzed using principal component analysis (PCA) with MultiExperiment Viewer (TMeV) software. Volunteers were examined in terms of tear production status (Schirmer I test), clinical assessment of palpebral lids and meibomian glands, and a subjective OSD questionnaire before tear collection by a glass micro-capillary.

**Results:**

Analysis of peptides and proteins in the 1–20 kDa range showed no significant inter-day differences in tear samples collected from six healthy individuals during seven days of monitoring, but revealed subtle intrinsic inter-individual differences. Profile analyses of tears collected from the right and left eyes confirmed tear bilaterality in four healthy patients. The addition of physiologic serum for tear sample collection did not affect the peptide and small protein profiles with respect to the number of resolved peaks, but it did reduce the signal intensity of the peaks, and increased variability. Magnetic beads were found to be a suitable method for tear film purification for the profiling study.

**Conclusions:**

No significant variability in tear peptide and protein profiles below 20 kDa was found in healthy controls over a seven day period, nor in right versus left eye profiles from the same individual. Subtle inter-individual differences can be observed upon tear profiling analysis and confirm intrinsic variability between control subjects. Addition of physiologic serum for tear collection affects the proteome and peptidome in terms of peak intensities, but not in the composition of the profiles themselves. This work shows that MALDI-TOF MS coupled with C18 magnetic beads is an effective and reproducible methodology for tear profiling studies in the clinical monitoring of patients.

## Introduction

Tear film is a complex gel mixture with a thickness of between 6 µm and 20 µm. It is distributed on the ocular surface and contains proteins, lipids, electrolytes, some small organic molecules and metabolites secreted by the main lacrimal gland and the palpebral accessory glands [1-3]. The normal tear volume is around 6 µl, with a mean secretion rate about 1.2 µl per min, and a turnover rate of approximately 16% per min [4]. In spite of being similar to other body fluids in terms of protein composition, tear film has a characteristically high concentration of proteins (8 μg/μl approximately), ions and antioxidant compounds [1,5], which makes it particularly suitable for proteomic analysis, despite the small volume of the tear.

In the past, tear film proteins have been studied by using gel electrophoresis and other techniques such as Edman degradation. These studies have revealed that the major tear proteins include lysozyme, lactoferrin, lipocalin, tear specific prealbumin, serum albumin, secretory IgA, and lipophilin [6-10]. More recently, almost 500 different proteins have been identified in the human tear film [5], although it has been estimated that 70%–85% of total secretory protein can be accounted for by lipocalin, lysozyme, lactoferrin and secretory immunoglobulin A [6,11]. The concentration of these major tear film proteins can oscillate between 1.5 to 2.07 mg/ml. However, the concentration of other low abundance tear film proteins is below 0.1 mg/ml [11].

In recent years, mass spectrometry (MS) based proteomics has been successfully used for the profiling study of different body fluids, such as urine [12], serum [13], blood plasma [14], and also tear [15]. This approach has several advantages over other proteomics methods, such as high sensitivity in the nanogram/picogram range, rapid performance, and the possibility of analyzing both protein and peptide abundance levels. Previous determinations and mapping of tear protein profiles have employed a variety of mass spectrometry technologies, such as surface-enhanced laser desorption ionization- time of flight (SELDI-TOF), matrix assisted laser desorption ionization-time of flight (MALDI-TOF) and liquid chromatography coupled with electrospray ionization (LC/MS) [16-19]. In particular, MALDI-TOF MS has been used to characterize low molecular weight protein masses [20] in the range of 848–3,897 Da, finding an absence of diurnal variation of low molecular weight species in tears.

However, the contribution to variability in tear protein studies by day-to-day and individual variability, as well as eye bilaterality remains to be characterized. Additionally, it has also been reported that differences in tear proteome information may arise from the method employed for tear sample collection, such as Schirmer test papers or capillaries [16,19]. Finally, it is currently unknown if the addition of physiologic serum for tear collection in the eye-flush method introduces additional variability in tear protein profiles with respect to standard methods [21,22].

Thus, in the present work we have studied variation due to tear sampling from the same individual on different days (inter-day variation) and due to different individuals (inter-individual variation), as well as variation due to sampling from different eyes of the same individual (inter-eye) in healthy controls using the standard capillary method. In addition, we evaluated differences in the tear film peptidome and low molecular weight proteins (in the 1–20 kDa range) measured by MALDI-TOF MS profiling due to capillary and eye-flush collection methods. Tear samples were enriched before MALDI-TOF MS by solid phase extraction (SPE) using reverse-phase affinity magnetic beads (C18) to enhance both reproducibility and the overall quality of the analysis. Finally, data exploratory analysis involving Principal Component Analysis (PCA) was used to interpret protein profile data and results were visualized using MultiExperiment Viewer (TMeV) software (Dana-Farber Cancer Institute, Boston, MA) [23,24].

## Methods

### Patient selection

The study was performed with 6 healthy volunteers, two men (32.50±2.12 years), and four women (33.75±10.24 years). The clinical characterization of these participants was conducted by medically qualified personnel. Written informed consent was obtained in accordance with the declaration of Helsinki on Biomedical Research Involving Human Subjects. Clinical features assessed included Schirmer test, examination of tear film stability, palpebral lids and meibomian glands. To confirm the absence of subjective symptoms, each patient was asked to fill out a modified Ocular Surface Disease (OSD) questionnaire about burning, itching, foreign body sensation, dryness and photophobia. Exclusion criteria included ocular surgery (two months before), allergy history, atopy, use of contact lenses, and administration of corticoids or any medication.

### Tear samples

Tear samples were collected with a 10 µl glass microcapillary (Micropipettes Blaubrand, Wertheim, Germany) from six control volunteer individuals. For tear sample collection, the microcapillary was placed close to the temporal lid margin of the eye, taking special care not to touch the conjunctiva.

To assess inter-day and inter-individual variability, tears were collected from all six control subjects (CT1-CT6) over seven consecutive workdays, without adding any type of lubricant or anesthesia (standard method). The bilaterality in tear samples was assessed in four volunteers (CT1, CT3, CT4, and CT6) from whom the tear sample was collected from both eyes using the standard method the same day. To study the effect of sample collection method on tear profiles, two weeks later four control subjects (CT1, CT3, CT4, and CT6) were selected for a four day monitoring. Each day, tears were collected from the left eye (LE) without the addition of physiologic serum (standard method), and from the right eye (RE) with a new glass microcapillary following previous addition of a drop of physiologic serum (eye-flush method).

For additional studies, a tear sample from CT5 was collected to perform solid phase extraction (SPE) assays, and additional tears from CT1 and CT2 were collected to verify the reproducibility of the magnetic bead technique for tear sample purification and enrichment.

Tear samples were always collected between 13:00–14:00 h, placed in 1.5 ml Protein LoBind Tubes (Eppendorf, Hamburg, Germany) spun down to remove the possible cellular debris and immediately stored at −80 °C until analysis. Tear samples were always collected by the same person, at the same time of the day, stored in the same type of tubes, and identically subjected to a systematic protocol of defreezing, processing and analysis, to minimize variability in results due to handling.

### Solid-phase extraction (SPE)

A purification step was included before MALDI-TOF MS analyses with the aim of enriching tear samples in peptides and low molecular weight proteins. We tested four reverse-phase based SPE methods: commercial and customized tips as well as magnetic beads.

Two types of homemade microcolumns were prepared. These consisted of Empore disks functionalized with octyl (C8) or octadecyl (C18) groups (3M, St. Paul, MN). Disks were manually inserted into pipette-tips. Commercial ZipTip Pipette Tips (Millipore, MA) equipped with C18 chromatographic media in the stationary phase were also tested. Finally, ferromagnetic nanospheres functionalized with C18 from MB-HIC18 Magnetic Bead Purification Kit (Bruker Daltonics, Bremen, Germany) were assessed.

In all cases, tear fluid samples were progressively thawed on ice, and subsequently 1 µl of each sample was diluted to 10 µl in MilliQ (Millipore, Bedford, MA) quality water. Purifications with C8 and C18 Empore disk microcolumns and ZipTips were performed by adding 5 µl of diluted samples (1:10) to micro-columns, followed by a washing step with 10 µl 0.1% trifluoroacetic acid (TFA). Sample elution was performed by adding 1 µl alpha-cyano-4-hydroxycinnamic (HCCA) matrix in acetonitrile: 0.1% TFA (70:30). Magnetic bead based purification was performed according to the protocol suggested by the manufacturer. After binding and washing steps, each purified sample was eluted with 5 µl of an ethanol:acetone (2:1) solution.

### MALDI-TOF mass spectrometry

For mass spectrometry, all tip preparations (Empore C8, C18, and Zip Tips) were performed by the dried droplet method (0.5 µl sample+0.5 µl HCCA matrix). In contrast, magnetic bead preparations were diluted (1:10) in HCCA matrix. In all cases, 1 µl of each diluted sample was spotted in quadruplicate onto an MTP AnchorChip 600/384 TF plate. Profile analyses were performed on an Autoflex III TOF/TOF Smartbeam spectrometer in which the profiling data were acquired in linear-mode geometry, using the following settings: ion source 1, 6.02 kV; ion source 2, 5.32 kV; lens, 3.01 kV. Ionization was achieved by irradiation with a solid state pulsed laser, operating at 66 Hz (30% attenuator). All spectra were obtained randomly over the spot surface (5,000 shots fired per spot) by an auto execute-method, in a mass range between 1 and 20 kDa. Profiling spectra were calibrated using the protein mixture Protein Calibration Standard I. All materials were from Bruker Daltonics (Bremen, Germany).

### Statistical analysis

Data Obtained from MALDI-TOF analyses were statistically analyzed with the ClinProTools 2.2 software (Bruker Daltonics), and all spectra were systematically processed as follows: baseline subtraction was performed by the Top Hat baseline algorithm to remove the broad structures of the individual spectra, facilitating peak selection based on signal/noise (S/N) ratio and intensity thresholds. All spectra were then normalized to their own Total Ion Count (TIC) and recalibrated to correct possible shifts due to the height profile of the preparation. From the recalibrated preprocessed individual spectra, a total average spectrum was calculated, as well as an average peak list representing all important peaks, which were used as features to determine statistical information. Data were also smoothened by the Savitsky Golay algorithm.

### Data representation

Due to the complexity of Mass Spectrometry data, different tools have been employed to facilitate data visualization and simplify their comprehension. Thus, data were represented in a gel view format, directly obtained from ClinProTools 2.2 software (Bruker Daltonics, Bremen, Germany). In this type of representation, the x-axis records the mass/charge (m/z) ratio, the y-axis displays the running spectrum (each with four technical replicates) and the peak intensity is expressed in arbitrary units (a.u.). In most cases, an average spectrum of each class was also included at the top of the gel view. This type of representation offers a view of the profile obtained, giving an idea of the technique reproducibility.

The statistical exploratory technique known as Principal Component Analysis (PCA) was used to reduce data dimension, diminishing the number of variables to a few principal components, containing the majority of variance. This pattern recognition method characterizes or separates studied samples into different clusters on the basis of their similarities, representing the results on a plot.

## Results

### Patients

A total of 6 healthy control individuals were included in this study. Schirmer I test values were higher than 5 mm/min in all cases. Fluorescein staining according to the Oxford Scale was grade 0 in all cases, and meibomian gland status was normal in all control volunteers. According to the modified OSD questionnaire, healthy subjects did not report any symptom related to eye disorder, such as sensations of burning, itching or grittiness in the eye, dryness, photophobia, use of ocular lubricants or eye drops, eyelid flaking, blurred vision, sleep, or sticky eyelids.

### Solid-phase extraction (SPE) system selection

**Figure 1:**
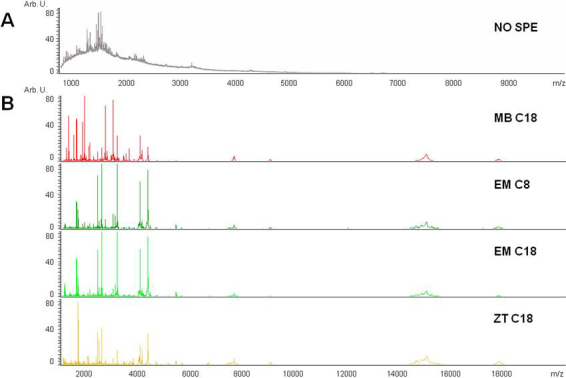
Mass spectrum of an unprocessed tear and a tear processed with solid-phase extraction (SPE) protocols. **A**: Direct analysis of a tear by MALDI-TOF MS produced a displacement of the baseline. This phenomenon is due to ion suppression produced mainly by the presence of salts and lipids. A limited peptide population can be noted. **B**: The same tear treated with four different SPE methods before MS analysis offers a spectrum which is more amenable for profiling analysis. MB C18: Magnetic Beads; EM C8: Empore Disc C8; EM C18: Empore Disc C18; ZT C18: Zip Tip.

First one crude tear preparation (1:10 water dilution) from patient CT5 was analyzed before evaluating the different SPE methods to examine the general profile associated with the tear mass spectrum. The obtained spectra indicated strong signal suppression, possibly due to the high concentration of salts and also lipids in tear (Figure 1A). However, when treated with four different SPE methods (C8 and C18 Empore discs, ZipTips and magnetic beads), the spectra showed a significant increase in global quality, with a high abundance of peaks in the peptide region up to 4 kDa and several masses up to 20 kDa (Figure 1B). This result clearly demonstrates the necessity of a pre-treatment for tear sample desalting and cleaning.

**Figure 2:**
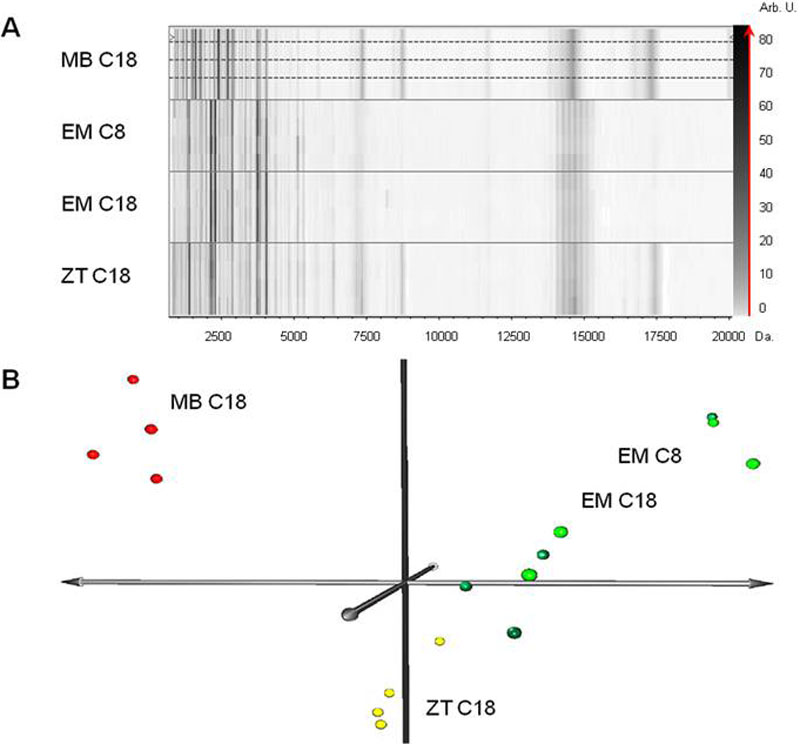
Proteome/peptidome profile gel view and principal component analysis (PCA) of tear samples purified with four different solid-phase extraction (SPE) methods. **A**: Virtual gel, representing the mass/charge (m/z) ratio (x-axis) of the detected peaks for each spectrum acquired (y-axis). The intensity of each peak is represented as a gray intensity scale corresponding virtually to the z-axis. In this gel representation, the same tear pretreated with four different SPE methods can be observed: Magnetic Beads (MB), Empore Disc (EM) C8, Empore Disc C18, and Zip Tip (ZT). In all the cases, each tear was spotted in quadruplicate (top profile) to verify the technique reproducibility and enhance statistical validity. **B**: PCA analysis showing that those samples treated with column-based systems (Empore C8 and C18) group together with higher dispersion, whereas the samples treated with magnetic beads and Zip Tip grouped separately and homogeneously.

Additionally, to select the optimal system for sample purification, 1 μl of tear from one patient (CT5) was purified with the 4 different SPE systems (Empore C8, C18, ZipTips, and magnetic beads). After purifying, the samples were analyzed by MALDI-TOF mass spectrometry under identical experimental conditions. This assay was repeated four times to evaluate the reproducibility of results. The peptide/protein profiles of each one of these preparations were obtained using ClinProTools 2.2 software (Figure 2). In general, a higher number of peaks were observed in the lower range of the spectra (below 4 kDa) with all the assayed methods. However, the pattern of peaks associated with C18 magnetic beads was distinct to those associated with the Empore and ZipTip methods. Thus, at a mass range <4 kDa, a higher resolution of peaks was observed when using magnetic beads. Next, we statistically determined the number of peaks provided by each technique, as well as the weighted harmonic mean of their coefficients of variation (CV%) in both the complete spectrum window (1–20 kDa), and in three ranges of different masses: 1–4 kDa, 4–10 kDa, and 10–20 kDa. Results obtained in terms of sensitivity (number of masses detected), and peak variations (intensity CV%) are summarized in Table 1. The number of masses detected and peak intensity variations (%CV) were obtained taking into account four technical replica per individual within the same sample preparation method, which subsequently were averaged.

**Table 1:** Tear sample purification methods.

** **	**Zip tip**		**Empore C8**		**Empore C18**		**Magnetic beads**	
**Mass Range (kDa)**	**peaks**	**CV%**	**peaks**	**CV%**	**peaks**	**CV%**	**peaks**	**CV%**
Total Spectra	102	14.93	84	15.96	81	28.04	104	11.00
Range 1; 1–4	55	14.37	52	16.14	49	34.74	73	9.25
Range 2: 4–10	36	15.85	20	14.78	24	20.62	20	24.92
Range 3: 10–20	11	14.96	12	16.25	8	25.49	11	19.87

Comparison of the efficacy of the four purification methods for enrichment of tear samples based on solid phase extraction. CV%, coefficient of variation.

When the total range is considered, both Empore disk based micro-columns (C8 and C18) rendered the lowest output, with only 84 and 81 peaks detected respectively, and relatively high variations in intensity (16 and 28%). The ZipTip based method provided an average of 102 masses with a CV% of 15%. The best overall results were obtained with the C18 magnetic beads, with the highest sensitivity (104 peaks) and the lowest CV% (11%).

Analysis of data spectra by PCA revealed that magnetic beads and ZipTips C18 rendered a good homogeneity in results, grouping the samples in spatial proximity, whereas the Empore Disks EM C8 and EM C18 systems presented a higher dispersion of data suggesting low reproducibility as illustrated in Figure 2B.

### Magnetic bead batch reproducibility

Based on these SPE results, C18 magnetic beads were selected and used for tear sample purification. Because two different batches were to be used for sample purification, a validation step was included to test bead batch reproducibility and thus, to discard any technical artifactual result on tear profile due magnetic bead batch variability. To this end, 1 µl of tear sample from patients CT1 and CT2 was processed with both batches (batches 1 and 2) from Bruker Daltonics, and analyzed by MS under exactly the same conditions as detailed above.

**Figure 3:**
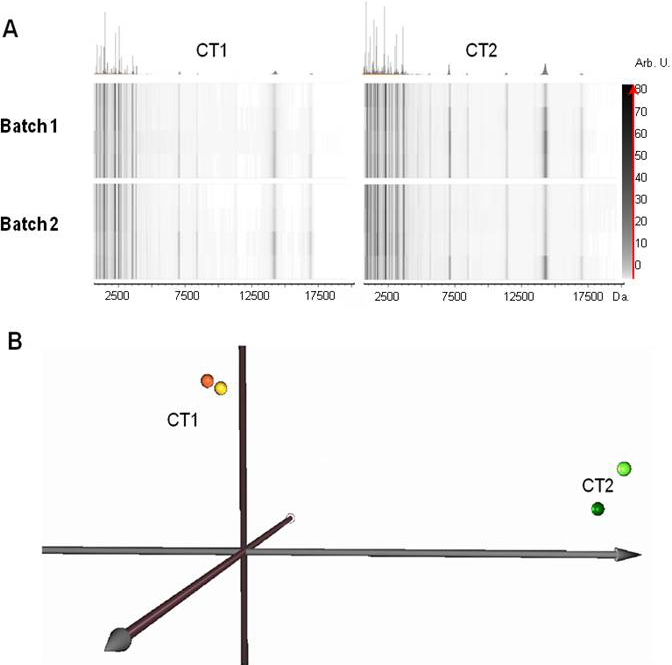
Batch reproducibility of magnetic beads used in this study for tear sample purification before MALDI-TOF MS analysis. **A**: Virtual gels representing profiles from control (CT) tear samples processed with two different magnetic bead batches (1 and 2) from the same company. Tear profiles were obtained after separately processing 1 µl of tear sample from control individuals CT1 and CT2 with both batches. The same number and intensities of peaks were observed in the profiles obtained from the CT1 tear sample treated with sphere batches 1 and 2. With tears from patient CT2, identical results were also obtained using both batches. These profile similarities indicate the reproducibility which can be obtained using the two tested batches. An inherent difference in peak intensities between patient CT1 and CT2 was observed due to inter-individual variability. **B**: The PCA plot identifies CT1 and CT2 as different individuals, which are spatially separated. However, sample from the same individual processed with bead batches 1 (light spheres) and 2 (dark spheres) from the same manufacturer, are almost coincidental.

The obtained profiles illustrated as virtual gels are shown in Figure 3A. CT1 and CT2 tear profiles showed slight differences in some peak intensities, due to inherent inter-individual variability. However, the individual profiles resulting from processing with either batch 1 or 2 exhibited high similarity among themselves, indicating reproducibility of the assayed batches. Reproducibility was found not only in peak numbers, but also in peak intensities, as can be seen in the virtual gels.

To more objectively corroborate this observation, PCA of the corresponding data was performed (Figure 3B). We found that CT1 and CT2 tears were clearly distinguishable on the PCA graph. However batch 1 and 2 data for each individual were practically coincidental, indicating that the profile resulting from treating the tear sample with batch 1 is quite similar to that obtained with batch 2. Thus, no differences were found due to bead batch, thereby corroborating the inter-batch reproducibility of magnetic beads.

### Inter-day monitoring of tear profile

**Figure 4:**
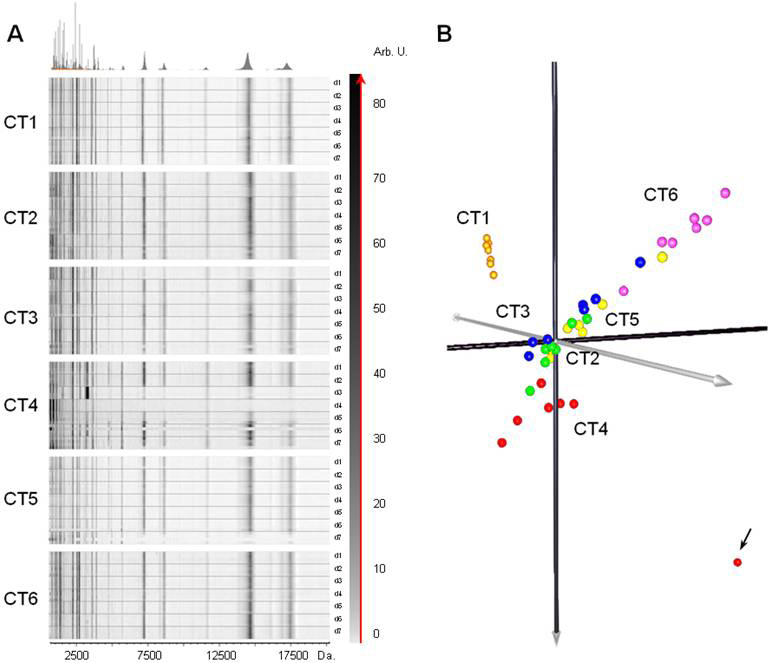
Monitoring of normal tear samples collected during seven consecutive days (day 1-7) from 6 control volunteers (CT1-CT6). **A**: Virtual gel views representing tear profiles appear to be similar from the first to the seventh day in each case. **B**: Principal Component Analysis (PCA) of analyzed tear profiles. PCA analysis confirmed that there is no significant variation of tear profiles from the same individual, and revealed outlier samples indicated by an arrow.

Six healthy volunteers were monitored over 7 days to evaluate inter-day tear profile variation. Tear samples were collected by the standard method once per day and analyzed by MALDI-TOF MS after purification. The resulting spectra exhibited an average of 96 peaks with a signal/noise ratio above 5 in a mass range between 1 and 20 kDa. We found that there was no significant variation in profiles of tears from the same patient (CT1) from day one to day seven. Similar profiles were also obtained for all other patients included in the study (Figure 4A), with only slight differences in peak intensities being apparent.

Representation of these results by a PCA (Figure 4B) revealed spatial proximity of samples from the same individual, indicating similarity in day-to-day tear profiles. We also observed a slight differentiation in samples from some controls, which is indicative of inter-individual variability. No gender or age effects were observed on the spectral profiles.

Tear spectra profiles of CT4 (days 3 and 4) were noticeably different to those obtained for the other monitored days (Figure 4A). This odd behavior can be attributed to technical variations during the processing of the samples, such as the different crystallization of the matrix over the Anchorchip in comparison to the rest of the spots. Consequently, PCA showed this effect as an outlier, mainly evident for day 3 which is completely separated from the group (Indicated by an arrow in Figure 4B). In spite of this shortcoming, neither the outliers, nor the replica have been excluded from the analysis, to give more robustness to the model.

### Tear film bilaterality

**Figure 5:**
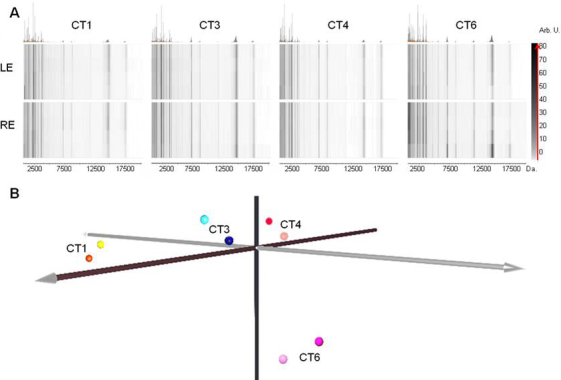
Analysis of tear film profile bilaterality in four control individuals. **A**: Virtual gel view of tear profiles obtained from volunteers CT1, CT3, CT4 and CT6 from left (LE) and right eye (RE). **B**: PCA analysis of the same tear profiles. An association and spatial proximity of left and right tear samples from the same individual is observed indicating similar profiles. A spatial separation of individuals CT1, CT3, CT4, and CT6 is also observed suggesting inter-individual variability.

To test the bilaterality of the tear profiles between right and left eye of the same subject, tears were additionally collected by the standard method from 4 control individuals randomly selected (CT1, CT3, CT4, and CT6) and subjected to MALDI-TOF MS analysis after sample purification. Results were illustrated as virtual gels (Figure 5A) and a PCA plot (Figure 5B). In all four cases, gel views indicated no significant differences between tears collected from the left and right eyes, indicating bilaterality of tears in healthy subjects. PCA confirmed this result, grouping samples according to the individual (CT1, CT3, CT4, or CT6), but not distinguishing tear source (left or right eye).

### Effect of sample collection method on tear film profiles

**Figure 6:**
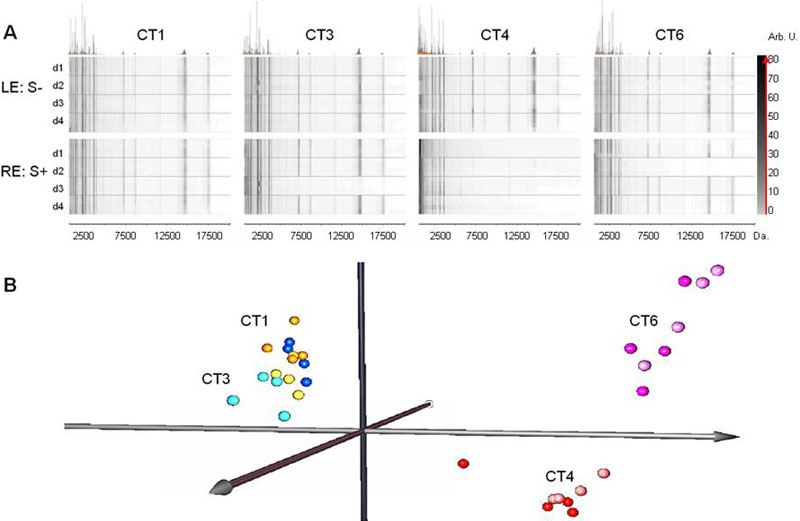
Effect of tear collection method on protein profiles. **A**: Gel view of tear profiles collected from CT1, CT3, CT4, and CT6 by standard capillary technique without addition of physiologic serum (LE: S-, left eye), and by the eye-flush technique involving the addition of physiologic serum (RE: S+, right eye). **B**: PCA representation of tear protein profiles obtained from the same individuals. Left eye tears were collected by standard capillary technique (light spheres) and right eye (dark spheres) by the addition of physiologic serum.

To evaluate if the sample collection method alters the outcome of the tear film profile of control subjects, tear samples from four volunteers CT1, CT3, CT4, and CT6 were collected from the right eye by means of the eye-flush method, and from the left eye without the addition of physiologic serum (standard method). These were then analyzed under exactly the same experimental conditions used for the rest of the samples in this study. Gel view spectra presented in Figure 6A suggested that similar tear film profiles are independent of whether physiologic serum was added or not for tear collection. However, a lower intensity peak signal could be seen in some profiles over 5 kDa with the eye-flush method. This weak peak signal, caused by tear dilution due to the serum, was notable principally in CT4, and also in profiles of CT3 (day 3) and CT6 (day 2). Nevertheless, when the spectra were subjected to PCA analysis, a clear association between samples from the same subject was observed, independent of the presence/absence of physiologic serum for tear collection, suggesting that despite weak intensities due to tear dilution, profiles were essentially maintained in the eye-flush tear collection technique (Figure 6B).

## Discussion

Proteins and peptides participate in the integrity and normal functioning of the tear film, contributing to protective and defensive roles, lubrication of the ocular surface and nourishing of the cornea. For these reasons, the study of peptides and light proteins has been gaining importance, since these molecules may reveal novel biomarkers and targets for therapy. Frequently, clinical trials find discrepancies which may be due to the inclusion of heterogeneous individuals in a specific group. This heterogeneity may distort results producing outliers. In the present work using MALDI-TOF MS, we have analyzed the tear profiles of healthy control individuals, and the possible effect on variation of parameters such as day-to-day subject variability, individual differentiation, and intrinsic differences in tears collected from the left and right eye. In addition, we evaluated the effect on profiling of physiologic serum addition for tear collection, as in the eye-flush technique, in comparison to the standard method of capillary-based collection.

Given the nature of the sample and the high sensitivity of MALDI-TOF technology, we took special care to standardize tear sample treatment for obtaining reproducible profiling data. The use of native, non-purified tear samples is associated with ion-suppression during MALDI-TOF analyses, due to non-peptide or protein contaminants such as salts, lipids and other compounds present in the tear. For this reason, contaminants must be removed to better resolve the sample by MS. A wide range of SPE methods with different chromatographic techniques is currently available [15,25,26]. Thus, we initially compared four different SPE methods for MALDI-TOF peptide/protein tear profiling. Our findings indicate that C18 magnetic beads are better suited for enrichment of tear samples in peptides and low molecular weight proteins in the range of 1–20 kDa. Moreover, for peptidome-oriented profiling studies of peptides up to 4 kDa, this method is particularly interesting because of the high number of peaks representing 70% of the total masses of the spectra which are present within this window. This treatment increased the quality of spectra, presenting an average of 96 peaks in a mass range between 1 and 20 kDa. These findings are corroborated by previous studies which have demonstrated the importance of human tear sample preparation before peptide/protein profiling by MALDI-TOF MS analyses [18,20,27], and the enhancement in peak detection that can be achieved when combinations with other enrichment methods are performed [28].

Many low-molecular weight proteins and peptides in human tears are potentially bioactive proteins. Using the technical approach which we report, it is possible to limit the study of the tear to peptides and proteins in the range below 20 kDa, thereby including some abundant proteins, such as lipocalin (17.5 kDa) and lysozyme (14 kDa), but discarding other abundant proteins such as lactoferrin (90 kDa) and secretory IgA (385 kDa). Additionally it would also be possible to improve the study of other smaller low-abundance proteins that might include promising biomarkers.

One of the reported disadvantages of magnetic beads for sample purification is inter-batch variability (even between batches from the same manufacturer), which can contribute to increased variability in results [27,29]. For this reason, we purified the same tear sample with two different magnetic bead batches, and analyzed the resulting fractions by MALDI-TOF MS. The results obtained indicated a high reproducibility between both batches, based on the number and intensity of peaks of the spectrum as shown in Figure 3. The superposition of profiles using PCA confirmed these results, and permitted us to conclude that at least in the present study, the magnetic bead batches used for sample purification give rise to reproducible results. We could thus rule out the possibility that variability in tear profiles might be due, at least in part, to bead batch variability. This finding provides further evidence that MALDI-TOF coupled with magnetic bead enrichment is presently an adequate method for proteomic and peptidomic profiling of tears.

The tear is a body fluid which is directly exposed to the environment and subjected to possible variations in its composition, regulation and stability. It has been reported for example that hormonal fluctuations, as well as circadian and nycthemeral rhythms can alter the flow and/or composition of the tear. Nycthemeral rhythms for example are responsible for more abundant tear secretion during the day versus the night. The brain limbic system also modifies basal tear secretion, which is lower in states of fatigue, anxiety and somnolence [30]. On the other hand, environmental factors such as air-conditioned workplaces, and occupational factors associated with decreased blinking in workers who use video or computer screens for long periods of time are factors which affect tear stability and consequently may contribute to rather high inter-individual variability in healthy control volunteers [31].

With a view to examining possible differences in the peptide/protein profile of tears due to intrinsic and extrinsic factors in healthy control volunteers, we analyzed fluctuations in tear profiles over a period of 7 days. To this end, healthy control volunteers were strictly selected in accordance with a series of clinical parameters, and tear samples were obtained under the same experimental conditions at the same time of the day. We compared not only the daily variation in the protein/peptide profiles of the tears of each individual during this time, but also the grouping of all individuals together as a hallmark of the homogeneity required in any control group. Our results confirm the tear profile homogeneity as a group, and also reveal the differences between the profiles of the individuals (as presented in Figure 4B). This observation let us to propose the use of such tear protein/peptide profiling technique for clinical trials to evaluate homogeneity of participants and identification of possible outliers.

Additionally, we also examined variability between tears from both eyes of a given individual, collected in the same way. To this end, we evaluated 4 controls and comparatively analyzed the profiles obtained for the right and left eyes. The profiles obtained (Figure 5) for the left eye strongly resembled those of its fellow eye, indicating that there are no significant differences in the tear profiles of the left and right eyes from the same control subject.

An issue which is currently unresolved is that of the most appropriate system for collecting tear samples. In 1994, Bjerrum and Prause [32] reported a method known as the “flush” tear collection technique which consisted in the instillation of a drop of physiologic serum into the eye to subsequently obtain the tear. Despite presenting clear advantages with respect to other systems, particularly in pathologies, such as dry eye, in which tear volume is reduced, it has been reported that the samples obtained with physiologic serum present inter-day differences and a high variability in tear protein levels [21]. For this reason, we evaluated the effect of the addition of physiologic serum during tear collection on tear protein profiles. To this end, once we had confirmed tear bilaterality in 4 healthy volunteers, we performed a comparative analysis in which tear samples were obtained from the same 4 patients during 4 days with the addition of physiologic serum to the right eye (flush tears) and without the addition of physiologic serum to the left eye (basal tears; Figure 6). This analysis showed that the addition of serum reduced in some cases the intensity of the peaks of the protein/peptide profile, principally in the range >5 kDa. Taking into account that sample purification was performed as a function of tear volume and not as a function of the total protein present in the tear, the observed signal reduction is surely due to the effect of dilution associated with the physiologic serum. Since the addition of serum varies the volume of the sample obtained from different individuals, a different protein concentration is obtained in the final volume to be analyzed. This fact is responsible for a large variability in the intensity of the profiles, not only between different individuals, but also between tears from the same subject obtained on different days. The variability is especially notorious in the case of the CT4, CT3, and CT6 controls, thus corroborating the observation of Ng et al. [21] who reported that physiologic serum addition for the obtaining of tear samples induces inter-day variability. Nevertheless, analysis of the profiles by PCA (Figure 6B) reveals a clear differentiation between samples as a function of the individual and not as a function of the tear collection system employed. The prior normalization of the intensities of each of the spectra by means of its own TIC (Total Ion Count) makes the effect of dilution non-significant when grouping samples with this type of tool.

Basal, reflex and flush tears from healthy non-contact lens wearers were recently compared, in terms of total protein content (TPC) and immunoglobulin A (IgA) concentrations, and the overall protein profile was established by gel electrophoresis (SDS–PAGE) and mass spectrometry (MS) [22]. These authors found that flush tears were significantly less concentrated than basal and reflex tears, and that the IgA concentration was higher in basal tears. However, the percentage of IgA with respect to total protein content in flush tears was not significantly different to that in basal tears. The authors concluded that flush tears present essentially the same spectrum of proteins in similar proportions, a finding which is corroborated by the present study. Nevertheless, our results additionally indicate that the tear-flush method can increase inter-day variability.

The fact that the spectrum pattern is not significantly altered by the tear-flush method may have special clinical relevance, since the addition of physiologic serum can facilitate tear collection and minimize the time required. This may be particularly relevant in pathological cases in which patients present a very low volume of tear, often making it impossible to obtain samples, as in the case of severe dry eye. In these cases, the addition of a drop of physiologic serum allows the obtaining of a manageable volume of sample for analysis, being more comfortable for the patient and less frustrating and time consuming for the researcher. Nevertheless, particular attention should be paid to the inter-day variability associated with the addition of physiologic serum and to the validity of determinations in studies in which a determination of absolute values (as concentration of proteins and peptides) is crucial.

In clinical studies involving tear samples, results are often found which cannot be explained by the clinical profile of the patient, deviating from the average behavior of the group. Several factors related with patient (environmental stressors) and sample processing (sample collection, material) may account for much of these biases. On the other hand, rigor in the methodology of sample processing is fundamental for obtaining reproducible results. For example, in studies of profiling, the variables introduced during processing such as making aliquots, working temperature, freeze-defreeze cycles, and final analysis of the samples contribute enormously to variability in the results [13,29,33,34]. In the present study, we have rigorously controlled these variables during the study.

In conclusion, this study demonstrates that MALDI-TOF MS coupled with C18 magnetic beads is a suitable methodology for tear profiling studies. The tear peptide and protein profiles which we obtained for control individuals testify to the absence of inter-day variability in samples from the same individual. In contrast, it is possible to observe inter-individual variability within the same control group. This facilitates the detection of potential outliers within a study which may lead to the distortion of results. Profiling analysis confirms the bilaterality of the tear from both eyes of the same individual, at least in healthy control individuals. Finally, we have shown that the effect of using the eye-flush method on the protein/peptide profile is not significant in terms of the number of resolved peaks; however, this method does significantly affect the intensity of the spectrum peaks and variability. The present study lays the foundation for subsequent studies involving tear analysis, not only in control individuals, but also in patients with pathologies which influence the tear.
